# Calibration-free benzene sensor for fire environments based on interband cascade laser absorption spectroscopy near 5 *μm*

**DOI:** 10.1007/s00340-025-08621-w

**Published:** 2026-02-21

**Authors:** Nicolas S. B. Jaeger, Yi Yan, Isabelle C. Sanders, Derek J. Urwin, R. Mitchell Spearrin

**Affiliations:** 1 Department of Mechanical and Aerospace Engineering, University of California, Los Angeles (UCLA), Los Angeles, CA 90095 USA; 2 Department of Chemistry and Biochemistry, University of California, Los Angeles (UCLA), Los Angeles, CA 90095 USA; 3 Los Angeles County Fire Department, Los Angeles, CA 90063 USA; 4 The International Association of Fire Fighters, Washington, DC 20006 USA

## Abstract

The toxicity of effluents from fires that occur at the wildland-urban interface is a growing concern among first responders, health professionals, and local populations. Benzene ($\hbox {C}_{6}\hbox {H}_{6}$) is a key fire emission of particular concern because of its high carcinogenicity. To better understand $\hbox {C}_{6}\hbox {H}_{6}$ production in harsh fire environments, this paper presents a calibration-free scanned-wavelength interband cascade laser absorption spectrometer for $\hbox {C}_{6}\hbox {H}_{6}$ that is demonstrated to be robust, portable, and accurate. The spectrometer targets a narrow-band absorption feature located near 2006 cm^−1^ ($\lambda _0 = 5~\mu$m). This wavelength selection is shown to be advantageous for measurements at near-ambient pressure conditions and to be well-suited for robust spectrally-resolved measurements with the limited scanning range of modern interband cascade lasers. The sensor employs a multi-pass Herriott cell to achieve an increased optical pathlength in a volume small enough to make the sensor portable for field measurements. The sensor is demonstrated to measure $\hbox {C}_{6}\hbox {H}_{6}$ over a wide dynamic range (sub-ppm to thousands of ppm) relevant to first-responder exposures. The method is also shown to remain stable over long durations of continuous logging and to effectively measure $\hbox {C}_{6}\hbox {H}_{6}$ content in structural fire effluents with varying ventilation conditions, accounting for potential interferers.

## Introduction

Recent natural disasters, such as the devastating Los Angeles fires of early 2025, have highlighted the dangers associated with fires that occur at the wildland-urban interface (WUI). A particular concern with WUI fires is their impact on community health via their effect on air and water quality [1, 2]. The potential scale of WUI fires, and their proximity to urban centers, presents the hazard of extremely dangerous air quality in localized areas. Aside from the community at large, which can suffer from the effects of poor air quality, there is a particular concern that first-responders may experience acute health risks due to their repeated exposures to more concentrated fire effluents. For many of the most hazardous species encountered by first-responders there is a scarcity of field-capable sensing systems that can provide timely insight into their exposure.

The challenge and importance of understanding toxic exposures associated with WUI fires has been exacerbated by the increasing heterogeneity of the associated fuel loads. WUI fires are those that occur where human made structures and developments meet undeveloped wildland fuels with a clear line of demarcation, and there exists high potential for structure to structure ignition. The fuel loads of WUI fires are thus much more complex, and potentially more dangerous, than those consumed by wildland fires where only vegetation burns. As urban areas develop, there is an increasing reliance on synthetic polymer materials in construction, infrastructure, vehicles, appliances, and contents such as furniture. The adoption of synthetic polymers greatly changes the emission profile of structural fires relative to wildland fires [3, 4]. The toxicity and prolonged impacts of large-scale urban fuel-loads is something that is not yet well understood and that requires further research [5]. State-of-the-art WUI models also need better quantification of emissions from structural fires than what is currently available [1]. Representative models of the material contents of urban structures, and the associated emission factors during combustion, are needed to better predict the toxicity of WUI fires. Detailed emissions measurements are key in validating such models, and sensors robust to smoke environments are critical for such measurements.

One of the key toxicants of concern from WUI fires is benzene ($\hbox {C}_{6}\hbox {H}_{6}$). Benzene is a minor product of most combustion processes [3] and is listed as a Group 1 carcinogen by the International Agency for Research on Cancer [6]. Human exposures to benzene are associated with multiple cancers of the lymphatic and haematopoetic systems such as acute myeloid leukemia and non-Hodgkin lymphoma [6]. There is currently insufficient information on how different fuel loads (particularly those including polymers) affect $\hbox {C}_{6}\hbox {H}_{6}$ production. Furthermore, the effect of different ventilation conditions on $\hbox {C}_{6}\hbox {H}_{6}$ production is not well quantified for most materials. A study by Hull et al. found that the production of many toxic species was highest when fires were under-ventilated [7], consistent with the expected rise in unburned hydrocarbons under conditions of oxygen deprivation. A better understanding of under-ventilated fire conditions is particularly important to the firefighting community because large-scale fires are generally under-ventilated [8]. A systemic review of studies investigating incidence(s), and mortality rate(s), of cancer among firefighters has shown that firefighters are at a significantly increased risk of developing, and dying from, cancer when compared to the general public [9]. Benzene is readily absorbed via inhalation, ingestion, and transdermal absorption. Meanwhile, firefighting personal protective equipment consists of textile ensembles with open interfaces. Thus, firefighters’ exposures to products of combustion such as benzene cannot be mitigated with the use of respiratory protection alone. Furthermore, several biomarker studies have demonstrated that firefighters are exposed to benzene when fighting fires, making measurements of this toxicant of great interest to the fire service [10–12].

Accurate emissions measurement systems are required to address the knowledge gaps in benzene production from fires. Furthermore, field deployable sensing systems that provide real-time, on-line feedback are desired to provide first responders with appropriate hazard assessments on a fire site. The United States Environmental Protection Agency (EPA) defines Acute Exposure Guideline Levels (AEGLs) for $\hbox {C}_{6}\hbox {H}_{6}$ as a function of exposure time. The eight-hour AEGL at which serious long term consequence can be expected (AEGL-1) is 9 ppm [13]. Likewise, the United States Occupational Safety and Health Administration (OSHA) defines the time-weighted average exposure limit of $\hbox {C}_{6}\hbox {H}_{6}$ to be 1 ppm over the course of eight hours or 5 ppm over 15 min [14]. Concentrations of $\hbox {C}_{6}\hbox {H}_{6}$ in weakly diluted smoke can be in the thousands of ppm in real fires. Concentrations this high are extremely hazardous, meeting the 10 min exposure limit set by the EPA for serious long-term health outcomes (AEGL-2) and the four-hour exposure limit at which death is likely (AEGL-3). There is a current lack of sensing systems that can quantitatively and selectively measure $\hbox {C}_{6}\hbox {H}_{6}$ over such a dynamic range in a harsh fire environment. Trace benzene detection in fire smoke is often measured with a gas chromatography-mass spectrometry (GC-MS) and/or flame ionization detection (FID) system [15, 16]. GC-MS and FID are highly precise and accurate. However, these systems are typically bulky, expensive, and slow. This means both methods are typically only feasible as an ex-situ measurement technique, requiring laboratory analysis of samples that are collected in canisters, bags, or sorbent tubes [15, 16]. Electrochemical sensors, such as those that employ metal-oxide semiconductors, are being developed that aim to address the size and cost concerns, however, these systems typically exhibit slow response times (on the order of minutes) and poor species specificity [17, 18]. Many of these sensing options also exhibit a small dynamic range, meaning they saturate easily. Optical methods such as open-path Fourier transform infrared (FTIR) spectrometers have been shown to achieve impressive detection limits (1–10 ppb) in wildfire smoke [19]. However, such systems require long and direct, free-space optical pathlengths on the order of tens to hundreds of meters, making them incapable of providing the highly localized measurements that are necessary to quantify personal exposure. Open path systems also always require two separate components (either a transmitter and receiver or a transceiver and retro-reflector) that require an unimpeded line-of-sight and that must be aligned in the field, hindering such a systems portability, accessibility, and deployment time. The spectral resolution of FTIR systems is dependent on mirror travel distance in the interferometer. While the spectral resolution of large FTIR systems can be very high, it is generally quite limited in any form-factor that would be amenable to a portable instrument. Recently, laser absorption spectroscopy (LAS) sensors have been demonstrated to be resilient in harsh fire environments and effective for characterizing emissions of toxic species such as HCN and CO [20, 21]. The demonstrated success of these sensors suggests that LAS may be an effective technique for measuring other fire effluents, including benzene. Recent works have shown laser absorption spectroscopy (LAS) to be a promising technique for characterizing $\hbox {C}_{6}\hbox {H}_{6}$ emissions with high specificity and accuracy, while also being feasibly portable [22–24].

This work describes a calibration-free mid-infrared laser absorption spectroscopy method for benzene detection suitable for robust detection and high dynamic range in fire environments. Following a brief theoretical discussion of scanned-wavelength LAS in Sect. 2, this paper presents a novel wavelength selection near 5 $\mu$m for $\hbox {C}_{6}\hbox {H}_{6}$ sensing that is shown to be advantageous for calibration-free near-ambient smoke measurements due to localized spectral structure that facilitates interference and baseline correction. Local high-resolution absorption cross-section measurements are shown to facilitate quantitative sensing over a range of conditions. The hardware design and digital signal processing framework are then presented with a focus on how key design choices allow for health-relevant detection limits in a relatively compact form factor using an interband cascade laser. A rigorous sensor validation is then reviewed in Sect. 4, in which the sensor detection limits, stability, and uncertainty are assessed. Finally, the laser absorption spectrometer is demonstrated to measure time-resolved $\hbox {C}_{6}\hbox {H}_{6}$ emissions from structure-scale fire effluents in the presence of heavy soot and interfering species. These large-scale fire measurements also demonstrate the field-readiness of such a sensing approach.

## Laser absorption spectroscopy

Molecules with either intrinsic or temporary dipoles can absorb incident electromagnetic radiation at specific frequencies associated with transitions in quantized internal energy. For a given molecule, the allowed quantum energy transitions are determined by its unique geometry and atomic composition, yielding a unique spectral absorption ‘fingerprint’ that can be exploited to determine various molecular properties. Laser absorption spectroscopy (LAS) is a non-invasive measurement technique in which electromagnetic radiation from a laser is passed through a sample of interest and the transmitted intensity is measured by a photovoltaic (PV) detector. Spectral absorbance can be measured from the ratio of transmitted to incident light intensity and related to various quantities such as species mole fraction, temperature, and pressure with precision and high-temporal resolution. The governing equation for LAS is the Beer-Lambert law,

1 $$\begin{aligned} \alpha (\nu ) = -ln\left( \frac{I_T(\nu )}{I_0(\nu )}\right) \, , \end{aligned}$$

where, $\alpha (\nu )$ is spectral absorbance of the sample, $I_T(\nu )$ is the spectral transmitted light intensity, and $I_0(\nu )$ is the spectral background light intensity [25].

The total absorbance of a sample can also be expressed in terms of the mole fractions and thermodynamic properties of its constituent species, as follows,

2 $$\begin{aligned} \alpha (\nu ) = \sum _{}\chi _iS_j(T)\phi _j(\nu , T, P, \chi )PL , \end{aligned}$$

or,

3 $$\begin{aligned} \alpha (\nu ) = \sum _{}n_i\sigma _i(\nu , T, P)L \, , \end{aligned}$$

where $\chi _i$ is the mole fraction of absorbing species, *i*, $S_j(T)$ is the linestrength of a specific transition, *j*, between energy states as a function of temperature, *T*, $\phi _j(\nu , T, P, \chi )$ is the spectral lineshape function of a transition as a function of temperature, pressure (*P*) and gas composition, $n_i$ is the number density of a species, $\sigma _i(\nu , T, P)$ is the spectral absorption cross-section, and *L* is the optical pathlength in the absorbing medium [25].

Localized absorption spectra are comprised of specific transitions or lines and their often convoluted lineshapes. Absorption lineshapes are a function of various broadening mechanisms, often dominated by Doppler and collisional broadening for infrared spectra, which are dependent on temperature and pressure. Desired quantitative properties from a sample can be derived by fitting the measured spectral absorbance with appropriate lineshape models. There are multiple models for these lineshapes, given as $\phi$ in Eq. 2. A common lineshape model, which was used for this work, is the Voigt lineshape [25, 26]. The Voigt lineshape is a convolution of a Gaussian function and a Lorentzian function that accounts for Doppler and collisional broadening, respectively [26]. For larger molecules with many transitions of similar energy, line-by-line fitting of measured spectra can be infeasible due to heavy overlap of many lines. In this case, a more empirically-determined absorption cross-section, $\sigma _i(\nu , T, P)$, can be used for fitting spectra.

By using a tunable laser source, the wavelength of incident light can be scanned across the target lines or absorption features by modulating the laser injection current. This provides spectrally-resolved information and makes measurements more robust to broadband fluctuations in light intensity when compared to fixed-wavelength LAS methods. Recent developments in laser technology have made spectra in the mid-infrared (mid-IR) more accessible with commercial low-power light sources, such as tunable interband cascade lasers (ICLs). The mid-IR is an advantageous regime to operate in because the energy of mid-IR light is well matched with the fundamental vibrational energy transitions of many molecules of interest, particularly hydrocarbons. However, for rapidly tunable distributed feedback semiconductor lasers, the current tuning range is typically only approximately 1 cm^-1^. It is, therefore, important to strategically target an absorption feature with enough narrow-band spectral content to be fully, or at least mostly, scannable by the chosen laser. This is necessary to realize the benefits of the noise- and interference-immune ratiometric measurements provided by scanned LAS techniques. The following section discusses the wavelength selection process for the $\hbox {C}_{6}\hbox {H}_{6}$ sensor presented in this work.

## Sensor development

### Wavelength selection

**Fig. 1:**
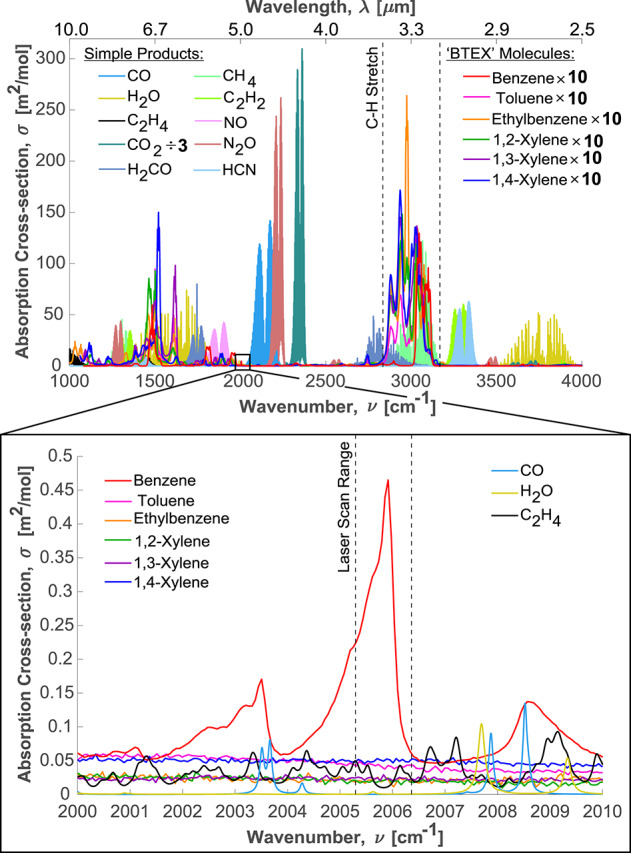
Broadband line survey of mid-IR cross-sections at 760 Torr (1 atm) and 298 K for likely species in synthetic fires (top) with a focus on the $\hbox {C}_{6}\hbox {H}_{6}$ feature targeted in this work (bottom) [30–33]

Following a comprehensive survey of the infrared absorption spectrum of $\hbox {C}_{6}\hbox {H}_{6}$, a feature located near 2006 cm^-1^ (5 $\mu$m) was selected for the laser absorption sensor. The target absorption feature, shown in Fig. 1, is likely attributed to an unassigned vibrational summation band of the $E_{2u}$ and $B_{2g}$ out-of-plane bending modes for C-H [27]. The asymmetrical profile of the feature is indicative of a vibrational bandhead with significant line blending and mixing [28, 29]. This feature is advantageous due to strong local differential absorbance and good isolation from other major combustion species. The selected wavelength is also accessible with low-power interband cascade lasers, which are key to portability.

Prior LAS works probing the infrared benzene spectra have targeted the C-H stretch band near 3000 cm^-1^ (3.3 $\mu$m) [22, 23] and the out-of-plane C-H bending mode near 674 cm^-1^ (14.8 $\mu$m) [18, 24, 34–36] with reasonable avoidance of interferers. Despite the high absolute absorption intensity at these bands, the high density of benzene lines renders very broad absorption features that span tens to hundreds of wavenumbers. These broad features often render very weak differential benzene absorption within any interference-free window spanning the approximately 1 cm^-1^ scan depth achieved by modern ICL lasers. This is particularly true at near-ambient pressures, mitigating the potential for scanned-wavelength calibration-free sensing that relies on local spectral structure to distinguish molecular absorption from window fouling, beam steering, and particle scattering among other broadband noise sources typical of harsh fire environments [37]. Moreover, in the case of the 674 cm^-1^ band, compact low-power tunable lasers suitable for highly portable sensor architectures are still an emerging technology and systems that target this region often rely on more complex difference frequency generation techniques [24, 34–36]. LAS studies of the C-H bending mode typically require liquid nitrogen cooled HgCdTe detectors to combat the thermal noise that is abundant at longer wavelengths. These detectors are not well suited for field-deployable sensors because they are often bulky and frequent refilling of the liquid nitrogen that they require is impractical.

**Fig. 2:**
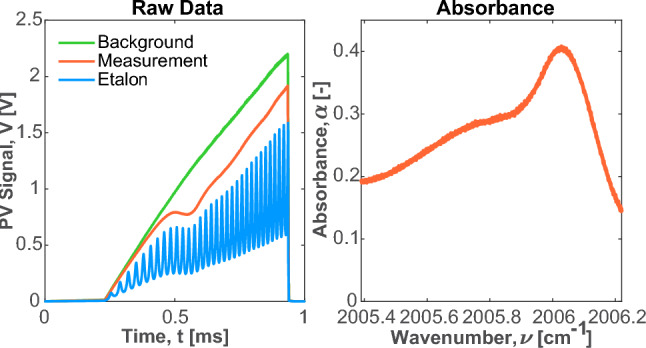
(Left) Sample PV detector data showing a non-absorbing background signal, an absorbing measurement taken from 535 ppm $\hbox {C}_{6}\hbox {H}_{6}$ calibration gas, and an etalon measurement that is used to establish a relationship between time and laser wavenumber. The measurements were performed at 25 $^\circ$C and 778 Torr with an extended 44.5 m pathlength. (Right) Associated spectral absorbance from the provided raw data

Figure 1 (bottom) shows that the $\hbox {C}_{6}\hbox {H}_{6}$ feature that was selected for this work has strong differential absorbance within the tuning range of a distributed feedback laser. The differential absorption is maintained at elevated pressure presumably due to line mixing associated with the densely packed lines within the vibrational bandhead [28]. Our prior work has similarly shown that targeting vibrational bandheads enables extension of pressure capability for robust scanned-wavelength LAS techniques [29]. In this application to smoke analysis, measurements nearer to ambient pressure reduce vacuum pump requirements, enhancing sensor portability. The typical scanning range for an ICL of approximately 1 cm^-1^ is insufficient to scan the entire feature, however, it is capable of capturing a significant portion of the $\hbox {C}_{6}\hbox {H}_{6}$ absorption spectra surrounding its peak absorbance. This is demonstrated in Fig. 2, which provides sample raw data for a background intensity measurement, a transmitted intensity measurement, and an etalon measurement for a calibration gas consisting of 535 ppm $\hbox {C}_{6}\hbox {H}_{6}$ balanced in nitrogen. Figure 2 also shows the associated spectral absorbance for the sample raw data, which is calculated with Eq. 1. As will be shown, the benefits of scanned LAS can still be realized, even with only a partially-resolved spectral structure, as long as accurate cross-section measurements are developed to fit the measured spectra.

The spectral isolation of the selected benzene feature is also shown in Fig. 1 with respect to other species that may be produced in fires. The only common combustion products that are expected to appreciably interfere with the $\hbox {C}_{6}\hbox {H}_{6}$ absorption feature are ethylene ($\hbox {C}_{2}\hbox {H}_{4}$) and water ($\hbox {H}_{2}\hbox {O}$). The interfering $\hbox {C}_{2}\hbox {H}_{4}$ features are attributed to the tail of the P-branch for the $\nu _6 + \nu _{10}$ combination band [31, 38]. The interfering $\hbox {H}_{2}\hbox {O}$ lines, located at approximately 2005.60 cm^-1^ and 2005.64 cm^-1^, are attributed to two different quantum states of the R(0,6) line for the H$_{2}^{~18}$O isotopologue of water [30]. The absorbance spectra of these interfering species are sufficiently isolated from the peak absorbance of $\hbox {C}_{6}\hbox {H}_{6}$ to be accounted for when processing the measured signal. As will be shown, the scanned-wavelength method allows for simultaneous measurement of these species to correct for any interference.

### Cross-section measurements

In order to enable quantitative interpretation of spectrally-resolved benzene absorption data at the target wavelength, high-resolution reference spectra, or cross-sections, are needed. Line-by-line models are typically preferred to account for variable thermodynamic conditions. However, the dense bandhead structure of the feature cannot be easily modeled with standard lineshape models because the individual constituent transitions are not well defined (i.e. a line-by-line database is not available). This complicates the ability to compensate for changes to sample conditions because a fitting algorithm cannot effectively solve for the spectroscopic parameters that define each line. Instead, complicated features such as that targeted here can be characterized by their absorption cross-sections as a function of temperature and/or pressure. This is achieved by measuring the absorbance of a sample at a given temperature and pressure and normalizing for species mole fraction and pathlength, as per Eq. 3. Of course, this requires that the sample thermodynamic condition be known from some other instruments for a particular measurement to be accurate. For this work, temperature and pressure measurements were attained with thermocouples and pressure transducers, which will be detailed in Sect. 3.3.

**Fig. 3:**
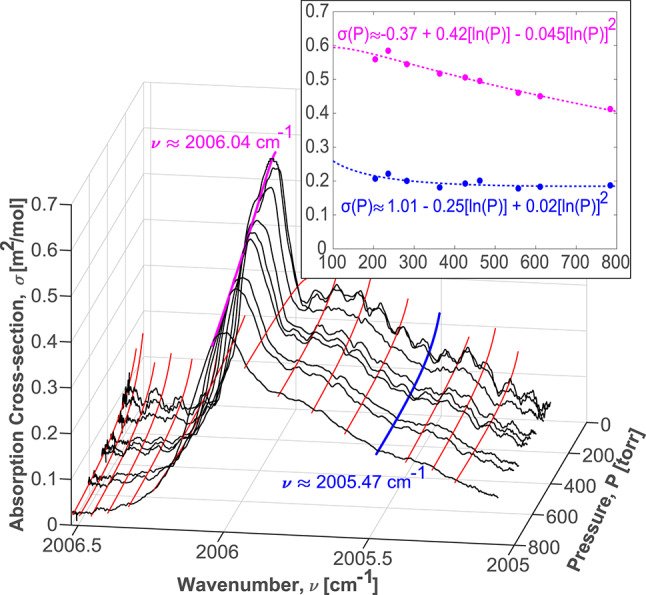
Measured $\hbox {C}_{6}\hbox {H}_{6}$ cross-sections with a visualization of how the pressure-sensitive second-order Boltzmann fitting protocol is applied across the spectral range

Through testing of fire effluents in smoke, it was observed that the sample temperature in the gas cell did not deviate by more than 3 K from room temperature due to requisite stand-off location of the sensor with respect to the fire source, which allowed the sample gas to cool. A study by Rinsland et al. included broadband cross-sectional measurements of benzene measured at three temperatures: 278 K, 298 K, and 323 K [32]. These cross-sections were used to perform an interpolated sensitivity analysis to quantify the effect of minor temperature fluctuations and to ensure that the constant gas temperature assumption was appropriate. The maximum estimated change in peak cross-sectional magnitude for a ± 10 K fluctuation in gas temperature is 6.6 %. As such, temperature-sensitive cross-sections were deemed unnecessary for this work. There was greater concern, however, that the sample pressure may drop in fire-conditions as a result of changing resistance across sample filters due to particulate accumulation in smokey environments. While it may be feasible to fix the pressure with appropriate regulation, flexibility in operating pressure was deemed important for sensor robustness. While the cross-sections in the Rinsland study were very useful in the wavelength selection process for this work, their utility for processing the data collected in this work was limited by the fact that they were only valid at 1 atm. The existing cross-sections were also measured by Fourier transform infrared spectrometry, which is limited by a relatively low spectral resolution (approximately 0.06 cm^-1^ in this case) [32]. It was, therefore, necessary to measure absorption cross-sections for $\hbox {C}_{6}\hbox {H}_{6}$ as a function of pressure with a high-resolution laser source. Cross-sectional measurements of $\hbox {C}_{6}\hbox {H}_{6}$ were performed from 2005.06 to 2006.52 cm^-1^ with a resolution of $1.5\times 10^{-4}$ cm^-1^ at 204, 236, 282, 363, 426, 462, 557, 611, and 784 Torr using the experimental apparatus described in Sect. 3.3, consisting of a 10.4 m optical pathlength. It has been shown that the Boltzmann statistics that govern lineshape and linestrength can be used to establish a relationship between absorption cross-sections and pressure and temperature [37, 39, 40]. A second-order Boltzmann fitting routine takes the following form,

4 $$\begin{aligned} \sigma (P,T)_{\nu }= & a_1(\nu ) + a_2(\nu )[ln(T)] \nonumber \\ & \quad + a_3(\nu )[ln(T)]^2 + a_4(\nu )[ln(P)]\nonumber \\ & \quad + a_5(\nu )[ln(P)]^2 + a_6(\nu )[ln(T)ln(P)], \end{aligned}$$

where the $a_n(\nu )$ terms are spectrally dependent coefficients derived from a least-squares fitting routine. These measurements were conducted at constant temperature, meaning Eq. 4 can be simplified to be just a function of pressure, as shown in Eq. 5

5 $$\begin{aligned} \sigma (P)_{\nu } = b_1(\nu ) + b_2(\nu )[ln(P)] + b_3(\nu )[ln(P)]^2 , \end{aligned}$$

where the $b_n(\nu )$ terms are a new set of fitted coefficients that include the *ln*(*T*) terms from Eq. 4. Figure 3 shows how this fitting routine is applied across the nine measured cross-sections.

**Fig. 4:**
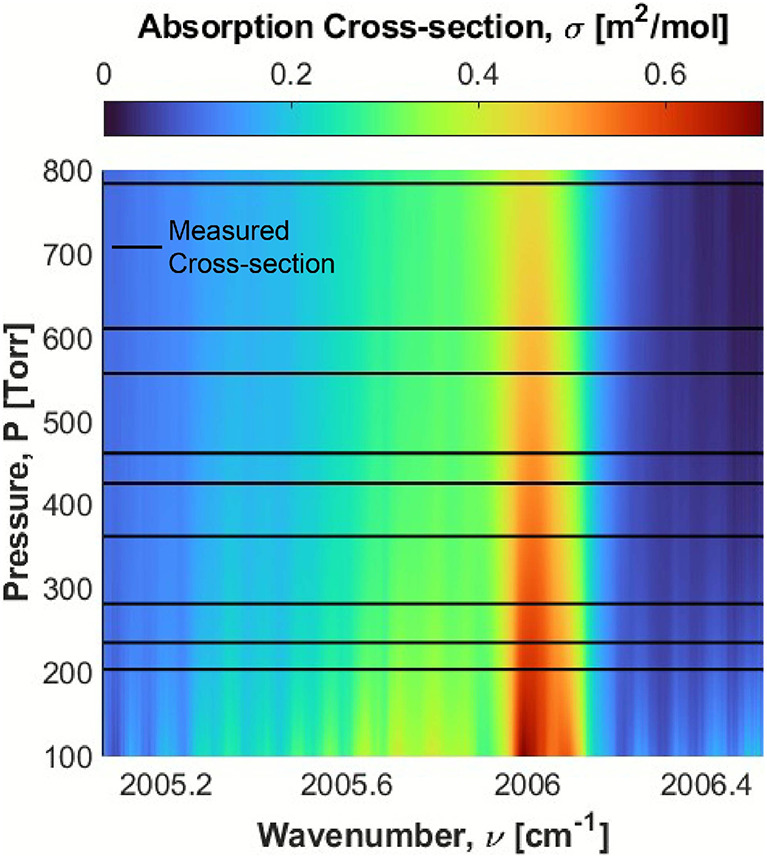
A heat map showing the full range of approximated cross-sections in 1 Torr increments based on the measured cross-sections, which have been marked as black lines

For the sake of clarity, Fig. 3 only shows 16 fitting algorithms, however, second-order Boltzmann fits of this form were applied to all 10,000 points in the wavenumber range to generate a pressure-based cross-section look-up table with high spectral resolution. The look-up table is defined from 100 to 800 Torr in 1 Torr increments. Figure 4 shows a graphical representation of the look-up table. Since cross-sectional measurements were only feasible in the range of 204–784, caution should be exercised when extending too far beyond this range.

Figures 3 and 4 show that the selected absorption feature maintains a relatively high differential absorption across all pressures, particularly around the feature’s peak near 2006 cm^-1^. As discussed in Sect. 3.1 this attribute makes this feature well suited for sensing with a soft vacuum or at ambient pressures. It can be observed from Fig. 3 that that the cross-sections at higher pressures appear relatively smooth, while those at lower pressures have distinct peaks across the spectral range. The low pressure cross-sections also have higher peak magnitudes. This is to be expected, as the constituent lines of the $\hbox {C}_{6}\hbox {H}_{6}$ feature exhibit less pressure broadening at lower pressures and, therefore, are more distinct. However, measurements made at a higher pressure will likely still exhibit stronger total absorbance, for a given concentration, than those made at a lower pressure, due to the larger number of absorbing molecules.

### Opto-mechanical design

**Fig. 5:**
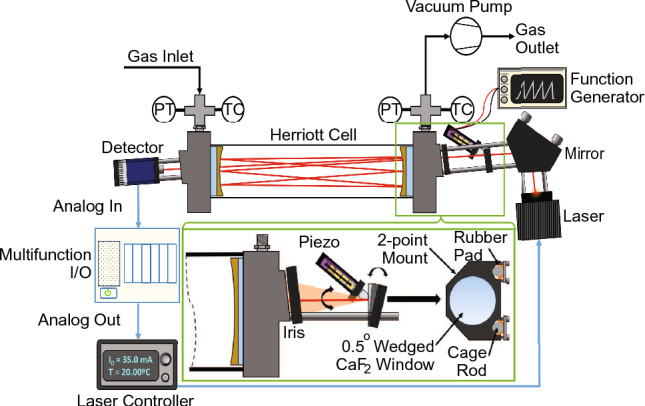
Schematic of the sensor’s hardware and sampling setup, with a focus on the piezoelectric dithering apparatus shown in the inset graphic

The spectrometer involves integrated opto-electronics and mechanical components for continuous measurements in a flow cell. A small Boxer 3KD series vacuum pump is used to draw sample gas through an optically accessible Thorlabs HC10L-M02 Herriott cell with a 10.4 m pathlength and 0.7 L sample volume. A Nanoplus distributed feedback interband cascade laser is used to access the selected absorption feature for $\hbox {C}_{6}\hbox {H}_{6}$. As shown in Fig. 5, the light from the ICL is pitched onto a planar silver mirror with three-axis control, such that it can be precisely directed through the optical input of the Herriott cell. After completing 28 internal reflections, the light then exits the Herriott cell via an optical output window and is measured by a Vigo Photonics PVI-4TE-5-1X1 photovoltaic detector. To save space and improve portability all of the optical components are mounted directly to the Herriott cell with standard 30 mm optical cage connecting rods. The optical components were contained in an aluminum enclosure measuring 27′′× 10′′× 6.5′′. This size represents a significant step in the process towards a more compact and turn-key system, as a single person can easily carry it on their own. There are also opportunities for further miniaturization in future iterations. A National Instruments PXIe-1073 chassis houses a PCI-6115 multifunction I/O unit that is used to record the photovoltaic signal produced by the Vigo detector to a Lenovo ThinkPad control laptop. The multifunction I/O unit is also used to generate an analog voltage which is translated into a modulated laser injection current via an Arroyo Instruments 6305 Laser Diode Controller. Miniaturized alternatives are commercially available for these outboard electronic components and they will be integrated into the optical enclosure in future designs. The multifunction I/O unit’s inputs and outputs are all controlled by custom LabVIEW software. An Evolution Sensors K-type thermocouple and an Omega PX309-015AI pressure transducer are installed on both the gas inlet and the gas outlet to measure sample temperature and pressure, respectively. The sensor also includes a piezoelectric dithering noise rejection system that is used in conjunction with digital data processing techniques to reduce optical interference fringes in the measured absorbance signal. This system is discussed in greater detail in Sect. 3.4.

### Signal generation and processing

An appropriate laser drive signal is necessary to achieve the desired temporal and spectral resolution for an LAS sensor. For this work, the laser is driven by a 1000 Hz sawtooth injection current function via the Arroyo Instruments laser controller. The modulation parameters of the injection current are governed by a modulating voltage signal that is supplied by an analog output of the multifunction I/O unit. The parameters of the sawtooth injection current and the laser chip temperature were adjusted to locate the $\hbox {C}_{6}\hbox {H}_{6}$ feature approximately midway in the spectral scan range of the laser. This prevents distortion that can arise due to edge effects that can exist at the start and end of the scan. The ICL temperature is maintained by a thermo-electric cooler (TEC), which is also controlled by the Arroyo laser controller. The TEC maintains a laser chip temperature of 20 $^\circ$C. A relationship between time and wavelength is derived from a 1-inch germanium etalon, which can be temporarily mounted in front of the PV detector in the sensor’s cage rod structure before or after a fire test. After some data cropping, the final usable scan range of the laser spans 0.81 cm^-1^, from 2005.41 to 2006.22 cm^-1^. At the start of the scan, the injection current is set to be below the lasing threshold of the ICL to allow for thermal dark current subtraction. The photo-voltage produced by the PV detector was sampled at a rate of 2 MHz by the multifunction I/O unit. The LabVIEW operating software performs on-line averaging of 1000 scans and then saves the resultant waveform to memory. The result is a single 2000-sample scan that represents a one-second time average of measured data.

Equation 1 shows how absorbance is calculated from the measured transmitted and background light intensity. For this work, the background signal was created by simply performing an LAS measurement of ambient air or nitrogen (when available). Air is primarily used in place of a non-absorbing background gas (such as nitrogen) for field measurements because it is more practical for the end use case as a portable measurement system. When air is used as the background the absorbance of $\hbox {H}_{2}\hbox {O}$ in ambient air is observed in the background signal, but it is accounted for in subsequent fitting routines. The dark current of both the background and transmitted signals is subtracted. The spectral absorbance is then calculated with an application of the Beer-Lambert law, given in Eq. 1. A least-squares fitting algorithm can then be applied to simultaneously derive the concentration of $\hbox {C}_{6}\hbox {H}_{6}$ and, in some cases, $\hbox {C}_{2}\hbox {H}_{4}$ and $\hbox {H}_{2}\hbox {O}$.

All optical systems are vulnerable to interference effects that occur due to the wave-like nature of light. The increased sensitivity that can be gained from the extended pathlength of a multi-pass Herriott cell can be reduced if a small percentage of the light is scattered from the primary beam and interferes with the primary beam at the detector. Such interference can result in wavenumber-dependent fluctuations in the measured output light intensity. Wavenumber-dependent fluctuations can also be caused by undesired, low-finesse, Fabry-Perot etalons (resonances) within the cell. A combination of such effects is expected to contribute wavenumber-dependent optical fringes in the measured light intensity. In some cases, the spectral width of the optical interference can occur on a scale comparable to that of Voigtian absorption features, which can hinder data processing capabilities. If the fringes are stable between the measured background and transmitted signals, its effect will not be observed in the calculated absorbance. However, minor changes in the optical alignment that can occur due to combinations of vibrations, beam-steering, and/or thermal expansion can cause the fringe to shift in wavenumber, which will affect the calculated absorbance and create undesirable noise.

**Fig. 6:**
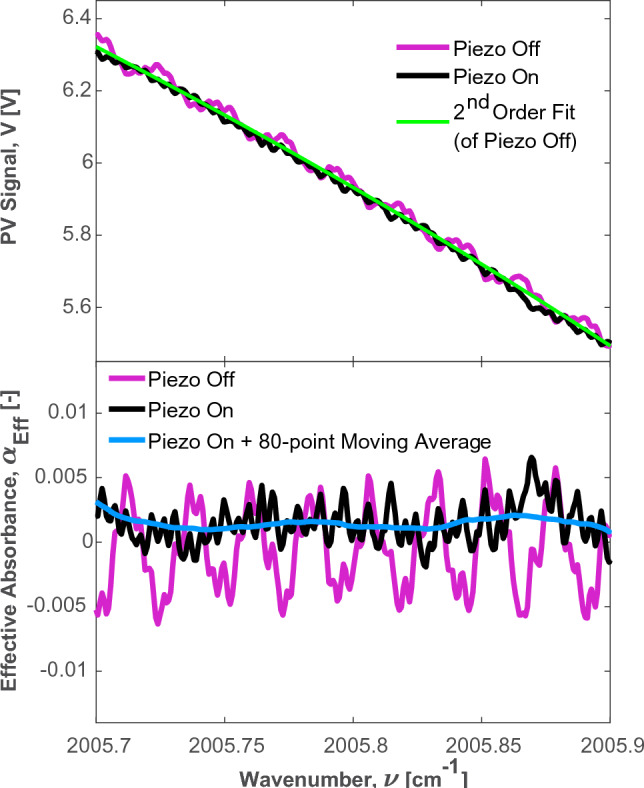
Demonstration of the effect of piezoelectric dithering on the raw PV signal (top) and the spectral absorbance when using an artificial background (bottom)

Robust opto-mechanical design can help reduce the effects of optical interference. It has been shown that the previously discussed fringe noise can be reduced mechanically by intentionally vibrating the optics on a time-scale faster than that of the time-averaging window. The vibrations must vary the total pathlength of the light by greater than $\pm \lambda /2$ [41, 42]. When the pathlength is modulated on this scale, the temporal location of the fringe pattern, as measured by the PV detector, will change from scan to scan. When multiple successive scans are averaged the shifting features will effectively cancel each other out, resulting in a smoother composite scan. This strategy is employed on the $\hbox {C}_{6}\hbox {H}_{6}$ spectrometer presented in this work. As shown in the inset graphic of Fig. 5, the light passes through a 0.5 $^\circ$ wedged calcium fluoride ($\hbox {CaF}_{2}$) window prior to entering the Herriott cell. The $\hbox {CaF}_{2}$ window is mounted on two of the optical connecting rods. The mounting points are fitted with rubber gaskets that allow for a slight rocking motion of the window about the mounting points. A piezoelectric stack (Thorlabs PK2FVP1) is positioned such that it contacts the window mount. A function generator (Rigol DG1032Z) is used to apply a modulating sawtooth voltage (0–10 V at 59 Hz) across the piezoelectric stack which causes it to expand and contract by approximately 6.7 $\mu$m. This causes the window to rotate slightly about the mounting points (approximately 0.014$^\circ$) which alters the angles of incidence and, therefore, the angles of refraction of the ICL beam transmitted through the $\hbox {CaF}_{2}$ window. Changing the angle of refraction slightly changes the input location of the light to the Herriott cell by approximately 10.8 $\mu$m, which results in a change in the pathlength traveled in the Herriott cell by a given photon. Calculating the precise change in pathlength is not trivial, however, it is experimentally determined that the change is sufficient to effectively cancel out to the most significant fringe noise when averaged over a one-second time window (1000 laser scans). A demonstration of this is provided in Fig. 6.

Figure 6 shows a portion of the PV data from a 1000-scan averaged data set. It is clear that when the piezoelectric stack is inactive the raw PV signal exhibits an optical fringe that appears as a noticeable oscillation in the measured voltage. This oscillation is clearly not as pronounced when the piezoelectric stack is turned on. An assessment of the effect of this oscillation in the absorbance domain is provided in the bottom portion of Fig. 6. As shown, an effective absorbance value is calculated from an application of the Beer-Lambert law (Eq. 1) in which the ‘Piezo Off’ and ‘Piezo On’ signals were used as the transmitted intensities ($I_T(\nu )$) and a second-order fit to the Piezo Off signal was used as the background signal ($I_0(\nu )$). There is clearly a noticeable oscillating signal with an effective absorbance of approximately ± 0.005 relative to the idealized background signal for the Piezo Off condition. When the piezoelectric transducer is engaged, these large oscillations are removed. However, there is still an noticeable oscillatory effect with a magnitude of approximately ± 0.001 that is either caused by higher order optical interference or noise. To achieve sub-ppm detection limits of $\hbox {C}_{6}\hbox {H}_{6}$ the spectrometer must effectively measure absorbance values on the order of $10^{-4}$ (depending on temperature and pressure of the sample gas), meaning that the residual noise observed in the Piezo On case must be mitigated.

**Fig. 7:**
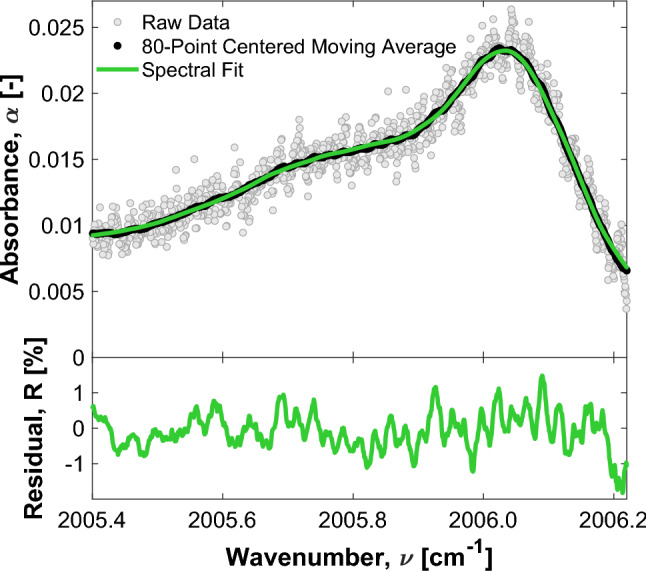
A sample fitting routine on 129.53 ppm $\hbox {C}_{6}\hbox {H}_{6}$ diluted in air, highlighting the effect of the moving-point filter on low-level etalon reduction

In instances where mechanical controls are insufficient to suppress higher order noise, digital signal processing can be used to further improve the signal quality. In this work, a centered moving average, which functions as a low-pass filter, was applied to the absorbance data. This is shown to effectively mitigate remaining unwanted fringes. For time domain signals, which LAS measurements effectively are given the relationship between time and instantaneous wavenumber, moving average filters are the optimal method for reducing noise and unwanted periodic behavior [43]. An adaptive approach was applied to the moving average filter, in which the number of points averaged was adjusted depending on the $\hbox {C}_{6}\hbox {H}_{6}$ absorbance. At lower absorbance values, when the relative magnitude of the residual interference patterns was more significant, more points were used in the averaging scheme. As many as 80 data points were considered in the averaging protocol. As shown in Fig. 7, the application of an 80-point centered moving average filter was applied along with the piezoelectric dithering to measure a mixture of $\hbox {C}_{6}\hbox {H}_{6}$ diluted in air. This served to validate that fact the window size would not harmfully distort the raw data, such that the fitting routine would not be able to accurately fit the processed data. Since the $\hbox {C}_{6}\hbox {H}_{6}$ feature is spectrally broad relative to the free spectral range of the resonances, the spectral content of its absorbance signal is shown to not be significantly impacted by the low-pass behavior of the moving average filter. An effective instrument function associated with the moving-point average was also applied to the fitted spectra for consistency.

## Sensor validation

### Lower detection limit

**Fig. 8:**
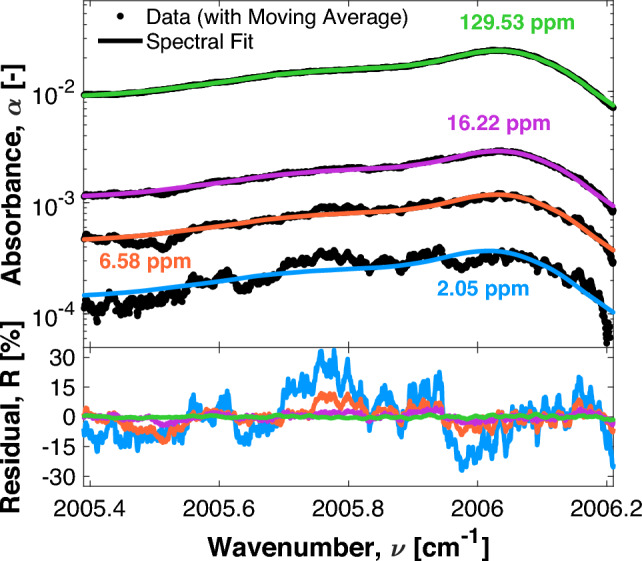
Sample data (with an 80-point centered moving average applied) at varying $\hbox {C}_{6}\hbox {H}_{6}$/air mixture ratios and their respective spectral fits and residuals

A single-ppm or sub-ppm detection limit for $\hbox {C}_{6}\hbox {H}_{6}$ is desired to align with the EPA and OSHA exposure limit guidelines for longer duration exposures. A gas mixing apparatus was created to achieve controlled concentrations of $\hbox {C}_{6}\hbox {H}_{6}$ for the purpose of determining the detection limit of the sensor. In this setup, 535 ppm $\hbox {C}_{6}\hbox {H}_{6}$ balanced in nitrogen was diluted with room air to create low-concentration mixtures of $\hbox {C}_{6}\hbox {H}_{6}$ in air. The dilution ratio of $\hbox {C}_{6}\hbox {H}_{6}$ was controlled by an MKS GE50A013203RMV020 mass flow controller. Manual valves were adjusted to maintain the sample pressure at approximately 752 Torr (as measured by an MKS Baratron 627B1BTDC18 absolute pressure transducer). Some representative sample data of low concentration scans from the gas mixing test, processed using the methods discuss in Sect. 3, are provided in Fig. 8 along with their respective spectral fits and residuals.

The limit of detection was determined by calculating the concentration at which the peak absorbance is equal to the minimum detectable absorbance (MDA) of the system ($MDA = 0.89 \times 10^{-4}$). For this work, the MDA was conservatively defined as the two standard deviation (2$\sigma$) noise across the spectral scan range of a low $\hbox {C}_{6}\hbox {H}_{6}$ concentration sample (the 2.05 ppm scan shown in Fig. 8). Based on this definition, a lower detection limit of approximately 0.50 ppm is inferred.

This detection limit is adequate for the lower end of the desired dynamic range, corresponding to long-term health-relevant benzene exposures, as defined by the EPA and OSHA $\hbox {C}_{6}\hbox {H}_{6}$ exposure limits (1–5 ppm) for extended periods. The 10.4 m optical pathlength can be adjusted to achieve a different lower detection limit with a trade-off in sensor size.

### Sensor stability

**Fig. 9:**
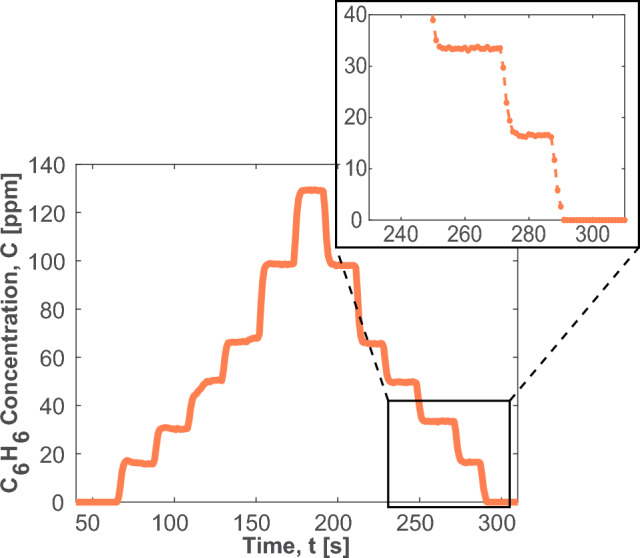
Staircase plot showing measured $\hbox {C}_{6}\hbox {H}_{6}$ concentration in $\hbox {C}_{6}\hbox {H}_{6}$/air mixtures

For prolonged use in the field, the sensor must also be stable over extended periods of time. The gas mixing setup was used to generate the staircase plot in Fig. 9, which demonstrates responsive measurements to controlled changes in benzene concentration over a period of approximately five minutes, with a focus on the lower end of the dynamic range (1–130 ppm) [44]. The inferred $\hbox {C}_{6}\hbox {H}_{6}$ concentration is demonstrated to be consistent for the same nominal flow rates of the diluted 535 ppm $\hbox {C}_{6}\hbox {H}_{6}$ mixture in both increasing and decreasing diluent concentrations. This evaluation shows negligible hysteresis in the measurement owing to the integrated calibration-free signal processing methods associated with the ratiometric scanned-wavelength technique which is immune to absolute signal drift. Finally, the sensor is shown to return to a 0 ppm baseline concentration when the $\hbox {C}_{6}\hbox {H}_{6}$ stream is cut-off, indicating no systematic bias or offset over the measurement duration.

### Uncertainty analysis

The uncertainty of the $\hbox {C}_{6}\hbox {H}_{6}$ measurements was determined by evaluating the uncertainty in the constituent variables in the Beer-Lambert law equations, as shown in Eq. 6.

6 $$\begin{aligned} \left[ \frac{\Delta \chi }{\chi }\right] _{C_6H_6}= & \bigg [\left( \frac{\Delta P}{P}\right) ^2 + \left( \frac{\Delta T}{T}\right) ^2 \nonumber \\ & \quad +\left( \frac{\Delta \sigma }{\sigma }\right) ^2 + \left( \frac{\Delta \alpha }{\alpha }\right) ^2 + \left( \frac{\Delta L}{L}\right) ^2\bigg ]^{1/2} \end{aligned}$$

Note that the uncertainty in number density for Eq. 6 is expressed in terms of uncertainty in pressure and temperature, as dictated by the ideal gas law. The uncertainty in pressure ($\Delta P/P = 0.0025$), temperature ($\Delta T/T = 0.0075$), and pathlength ($\Delta L/L = 0.0014$) were all manufacturer specified uncertainties for the pressure transducers, thermocouples, and Herriott cell, respectively. The peak absorbance of the $\hbox {C}_{6}\hbox {H}_{6}$ fitting routine and the MDA were used to assess the uncertainty in absorbance ($\Delta \alpha /\alpha = MDA/max(Fit)$).

The average uncertainty in the $\hbox {C}_{6}\hbox {H}_{6}$ cross-sections ($\Delta \sigma /\sigma$) was quantified to be 0.049. To assess this, the robustness of the quantitative interpretation used to derive pressure-specific cross-sections from the nine measured cross-sections was tested. This was done by sequentially removing one of the seven measured cross-sections from the middle of the data set and then comparing the percent difference between the approximated cross-section and the removed cross-section, as shown in Eq. 7.

7 $$\begin{aligned} \frac{\Delta \sigma }{\sigma }\bigg |_{P} = \frac{1}{n}\sum _{i=1}^{n}\bigg |\frac{\sigma (P,\nu _i)_{approx}\!-\!\sigma (P,\nu _i)_{meas}}{\sigma (P,\nu _i)_{meas}}\bigg |\, \end{aligned}$$

The average variation of these seven tests was then taken as the uncertainty in the cross-sections. The average uncertainty in Fig. 9 is 5.4 %, with a maximum uncertainty of 24.7 % for the minimum achieved concentration of 2.05 ppm.

## Benzene sensing in fire effluents

### Accounting for interfering species

In measuring benzene at the target wavelength in fire effluents, neighboring $\hbox {H}_{2}\hbox {O}$ absorption must usually be accounted for, while for some fuels and highly underventilated conditions, $\hbox {C}_{2}\hbox {H}_{4}$ absorption lines were also present and required accounting. Here, the methods for simultaneously fitting and correcting for these two potentially-interfering species are described. It should be noted that for these species, line-by-line databases are available. Table 1 provides a list of the relevant $\hbox {C}_{2}\hbox {H}_{4}$ and $\hbox {H}_{2}\hbox {O}$ lines that were considered in the fitting routine used in this work.

**Table 1:** Spectroscopic parameters of the interfering $\hbox {C}_{2}\hbox {H}_{4}$ and $\hbox {H}_{2}\hbox {O}$ lines considered in this work [30, 31]

Species	*ν*_*ij*_	*S* (296 K)	E′′
	[cm^-1^]	[cm^-1^/(molecule · cm^-2^)]	[cm^-1^]
C_2_H_4_	2005.272	5.573E-23	475.30
C_2_H_4_	2005.285	5.573E-23	475.32
C_2_H_4_	2005.328	3.812E-23	129.11
C_2_H_4_	2005.527	1.410E-22	412.55
H_2_O	2005.603	2.545E-24	880.11
H_2_O	2005.644	7.606E-24	880.08
C_2_H_4_	2006.136	1.074E-22	450.66
C_2_H_4_	2006.295	6.029E-23	429.75

**Fig. 10:**
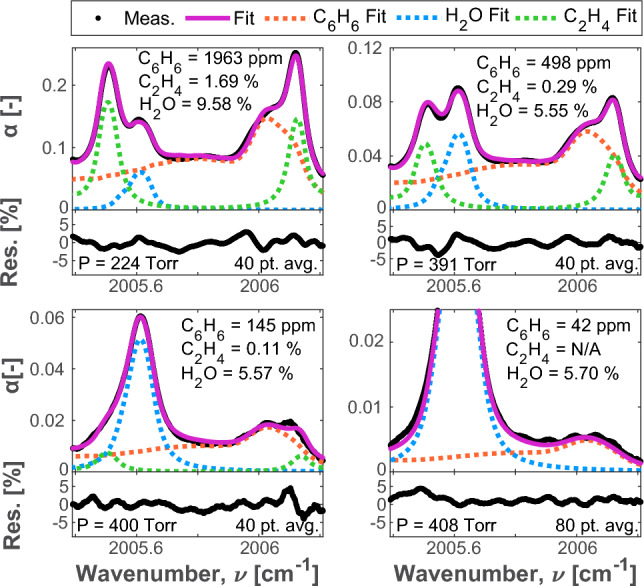
Four sample spectral fitting routines from real fire effluents, showing the dynamic range of the sensor. The total fits are shown as well as the fits of the constituent species: $\hbox {C}_{6}\hbox {H}_{6}$, $\hbox {C}_{2}\hbox {H}_{4}$, and $\hbox {H}_{2}\hbox {O}$. A 40-point centered moving average is applied to the plots containing 1963 ppm, 498 ppm, and 145 ppm $\hbox {C}_{6}\hbox {H}_{6}$. An 80-point averaging scheme is applied to the remaining plot with 42 ppm of $\hbox {C}_{6}\hbox {H}_{6}$. There is no contribution from $\hbox {C}_{2}\hbox {H}_{4}$ shown in the bottom right plot because the $\hbox {C}_{2}\hbox {H}_{4}$ emissions were below the detection limit for the sensor

The six $\hbox {C}_{2}\hbox {H}_{4}$ lines and the two $\hbox {H}_{2}\hbox {O}$ lines are fit with Voigt profiles, as per Eq. 2 [26]. Equation 3 is simultaneously applied to fit the $\hbox {C}_{6}\hbox {H}_{6}$ feature with the pressure sensitive cross-sections shown in Fig. 3. Leveraging the scanned-wavelength method, a linear baseline is also included in the fitting routine to account for low-frequency instabilities in the laser’s output wavelength that may occur as a result of temperature changes and laser drift [45, 46]. This also accounts for broadband intensity fluctuations that can occur because of beam-steering, scattering, or sensor fouling, creating a more robust sensor that does not require calibration. Furthermore, this also helps account for any broadband attenuation that may be caused by the tails of absorption features from minor interferers, which may be hard to de-convolve over the scan range. Such minor broadband interferers include the other ‘BTEX’ molecules (toluene, ethylbenzene, and xylenes), which are highlighted in the bottom panel of Fig. 1. Four samples of fitted spectral absorbance from fire effluents with varying gas concentrations are provided in Fig. 10 to highlight a range of sensor operability and interference at varying real smoke conditions. The moving average filter is applied to the data sets and shown not to significantly impact the ability of the data to be fit with the Voigtian lineshapes. The top subplots of Fig. 10 capture specific cases where the ethylene mole fraction has been pronounced ($\sim$1%) by measurements sampled very near the fire source at highly underventilated conditions. Despite the exaggerated interference, such conditions also tend to amplify the benzene mole fraction, providing ample absorption for a multi-spectral fit and highlighting the upper end of the demonstrated dynamic range. At conditions further from the peak flame intensity, ethylene decreased significantly in smoke relative to benzene, presenting less of an interference concern (see lower subplots).

### Benzene measurements in structural fires

**Fig. 11:**
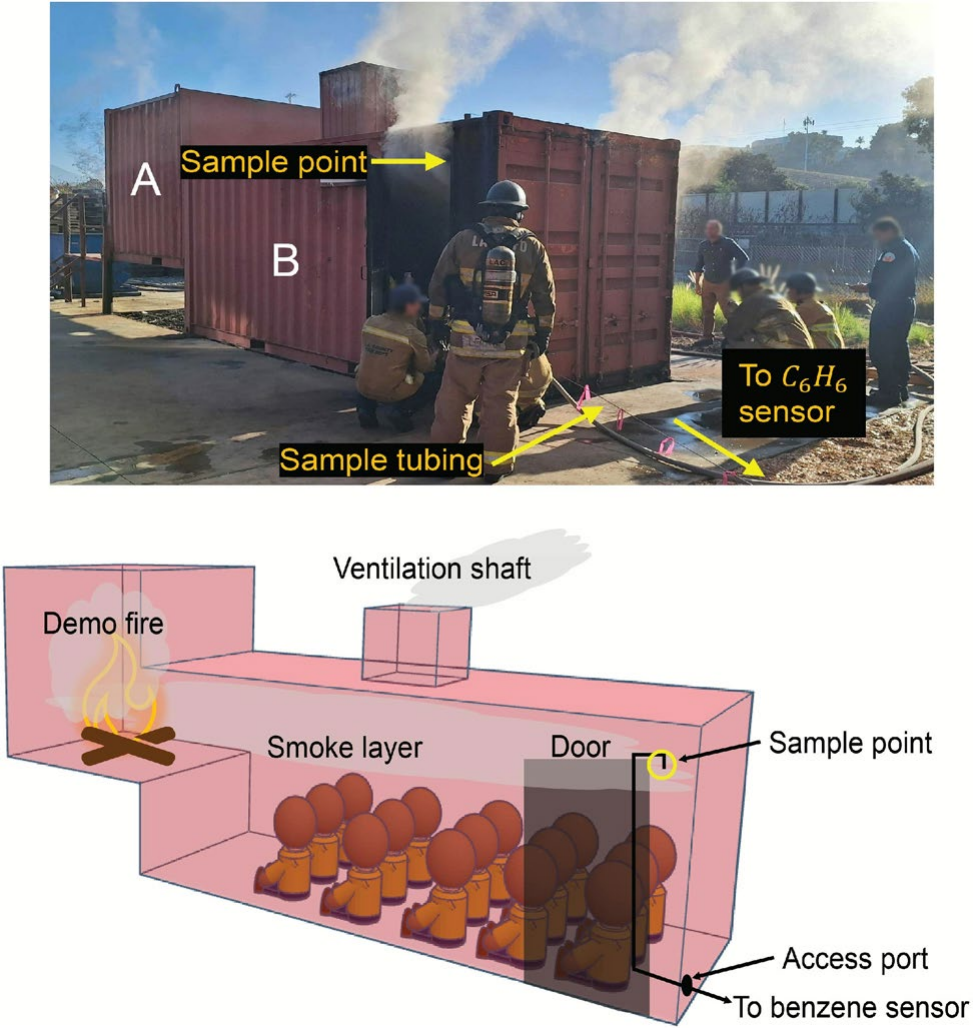
Structure-scale fire experiment setup at LACoFD East Valley training center

To validate the sensor’s ability to measure $\hbox {C}_{6}\hbox {H}_{6}$ in harsh fire environments for prolonged periods, emissions from structure-scale fires were measured through a partnership with the Los Angeles County Fire Department (LACoFD). LACoFD operates a training center in Pomona, California for prospective fire fighters to learn about fire dynamics and to practice fire suppression techniques. One of the facilities at the training center is a structure intended to teach trainees about the effects of ventilation on fire dynamics. The structure, shown in the top panel of Fig. 11, is constructed out of two shipping containers. The elevated shipping container (Container A) is a half-length container in which a wood-burning fire is ignited. The walls of Container A are also lined with an acoustic insulation board, which eventually catches fire as well. The full-length shipping container (Container B) is located at ground level and is joined to Container A. The intermediate wall between the two containers is cut away, such that the fire in Container A is visible from Container B. The smoke from the fire in Container A also diffuses into Container B, creating a smoke layer. As shown in the bottom panel of Fig. 11, prospective fire fighters sit on the floor of Container B during a training session (approximately 20 min in duration) and observe the progression of the fire. During a test fire, course instructors will alter the ventilation state of the fire by opening or closing the doors on either container to simulate events that may be observed on the fire site, such as flashover. In addition to exposures at WUI fires, firefighters seek to minimize exposure to products of combustion at all types of fires, including training fires that occur in controlled environments. Fent et al. demonstrated that firefighters participating in live fire training events exhibited a 2 to 7 fold increase in exhaled breath benzene immediately following the training fire [11]. Hence, these events presented not only an opportunity to evaluate the sensor, but to measure toxic exposures of interest to firefighters.

Emissions from a demonstration fire were sampled via stainless steel tubing within the smoke layer. The tubing was positioned inside Container B on the opposite end of the container from the source fire. The sample point was located near the ceiling of the container, such that it would sample from the smoke layer. The tubing was bent downwards to prevent excess soot deposition in the tube that could cause it to clog. The sample line extended to the bottom of Container B, where it could pass through an access hole drilled into the back wall of the container. The sample line then extended approximately 20 feet to a table, on which the benzene sensor was stationed. The sensor has two needle valves connected to the gas inlet. One is connected to the sample line located in Container B and the other is left unattached, such that it may allow ambient air into the Herriott cell. Prior to the start of a fire, the inline filters on the sample line were cleaned. The valve to the container sample line would then be closed while the needle valve to atmosphere would be left open to sample ambient air. This would help ensure the cell is purged of any particulate or adsorbed species prior to the next fire. During this process the multifunction I/O unit would continuously log 1000-scan averaged raw intensity measurements from the PV detector. Upon receiving notification from LACoFD personnel that the next fire was about to begin, a background measurement was performed using ambient air. The needle valve to atmosphere was then closed and the valve to Container B was opened. The multifunction I/O would then continue to save 1000-scan averaged signals from the PV detector to the control laptop for the duration of the fire, providing a 1 Hz effective measurement rate. Following the fire, the sensor would continue to sample air from Container B and the data would be saved accordingly to capture any residual $\hbox {C}_{6}\hbox {H}_{6}$ that may linger in the container. Due to time constraints associated with the training schedule, the measurements would be halted approximately 20 min after the fire was extinguished in order to have sufficient time to reset for the subsequent fire.

**Fig. 12:**
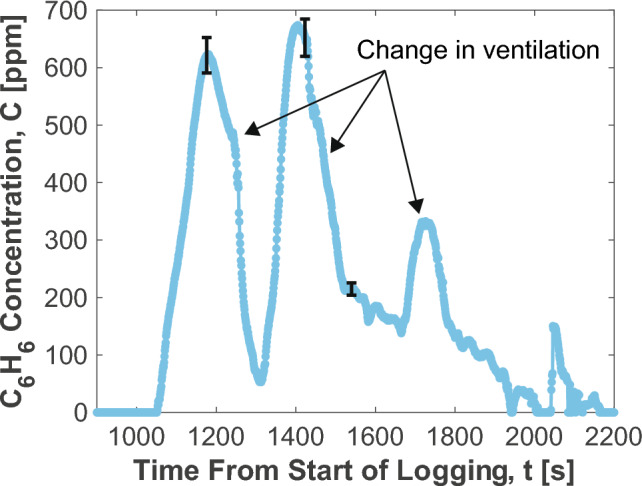
Time history of $\hbox {C}_{6}\hbox {H}_{6}$ measured during a representative structure-scale fire at LACoFD with relevant ventilation states highlighted

Following the processing routine outlined in Sect. 4, the $\hbox {C}_{6}\hbox {H}_{6}$ time history pictured in Fig. 12 was attained for a representative container fire experiment. A video of the fire was recorded to allow for a comparison of key events with the post-processed data set. It was observed that the duration of the peaks in the measured $\hbox {C}_{6}\hbox {H}_{6}$ production roughly aligned with the amount of time during which the main door of the training facility was closed. This is consistent with $\hbox {C}_{6}\hbox {H}_{6}$ production being higher in under-ventilated conditions. Precise time synchronization of events is complicated by the transport time required for the fire effluents to reach the sample point at the end of the container and the latency associated with sampling lines. The transit time of gas in the sample tubing was approximately 0.5 s/ft, as estimated in the laboratory. In addition to ventilation, other factors may influence benzene production including the evolving state of the fuel load over time, its temperature, char formation, and total percentage burned, which could not be assessed by the research team in this preliminary experiment due to practical constraints, but may be assessed in future work. It is also noteworthy that $\hbox {C}_{6}\hbox {H}_{6}$ signal exhibits good stability at 0 ppm before and after the test period. This reaffirms the results of the laboratory experiments discussed in Sect. 4 indicating that the sensor does not experience significant drift over long time periods. It is also notable that the dynamic range of the sensor was well-suited to this realistic fire experiment, capturing the full range of concentrations exhibited in the smoke layer from single ppm to above 600 ppm.

## Conclusion

This work presented a calibration-free, scanned-wavelength interband cascade laser absorption spectroscopy method for benzene measurements in fire effluents. Novel wavelength selection targeted a bandhead feature near 5 $\mu$m that involved minimal interference and strong differential absorption within the tuning range of a compact, low-power distributed feedback ICL. Benzene absorption cross-sections were measured over a range of pressures to enable quantitative inference of mole fraction with variable sample conditions. The sensor was validated in laboratory experiments, using a combination of optical fringe suppression with a piezo element and digital signal processing to achieve a lower detection limit of approximately 0.5 ppm with excellent stability and 1 Hz measurement rate. Multi-spectral fitting methods were shown to take advantage of the spectrally-resolved measurement to mitigate water and ethylene interference, preserving the calibration-free nature of the measurement over a wide dynamic range, with an upper limit exceeding 1900 ppm. The sensor was demonstrated to assess benzene dynamics during live fire training with the Los Angeles County Fire Department, reinforcing sensor stability and robustness over extended durations and the ability to capture relevant benzene levels in a realistic structural fire smoke layer with varying ventilation. The spectrometer is expected to facilitate analysis of benzene in wildland and structural fire effluents to better understand exposures of first responders and local communities impacted by fires.

## Data Availability

No datasets were generated or analysed during the current study.
